# Supplementary material for: Learning curve and surgical outcome of robotic assisted colorectal surgery with ERAS program
Source: Sci Rep. 2022 Nov 29;12:20566. doi: 10.1038/s41598-022-24665-w (PMC9709162; doi:10.1038/s41598-022-24665-w)
Supplement: Supplementary file 2 — Supplementary Information 2. [file 41598_2022_24665_MOESM2_ESM.zip › RAL Raw data-Ñ[▒K/082/HS_ñw╝╨░O▒Kñσ.pdf]

正式摘要(電子簽章：000000217116140)<br>W95 / W95 英文標題(注意:無英文姓名-請補病人基本檔!)	【讀取】憑證	

臺中榮民總醫院

出院病歷摘要

轉入號 : 轉出號 :

[redacted]

(8)流水編號	
(9)入院日期	110/05/12 (2021) 直腸外科 W95 - 35
(10)轉科日期	
(11)出院日期	110/05/17 (2021) 住院天數計 5 日

診斷

- 入院 :A-coon polyp
  PH:
  1. HTN under medications
  2. Anxiety under medications
  3. HIVD of L5 s/OP in 2010
  4. Right breast s/p OP in 2020
- 出院 :A-coon polyp
  for Robot Assisted right hemicolectomy on 2021/05/13
  PH:
  1. HTN under medications
  2. Anxiety under medications
  3. HIVD of L5 s/OP in 2010
  4. Right breast s/p OP in 2020

主訴

- admitted for Robot Assisted Colectomy.

病史

- The 61-year-old female had underlying disease of
  1. HIVD L5-S1, s/p Discectomy on 2010/04/22.
  She received colonscopy this year at LMD and revealed colon polyp, sessil. Therefore, this time, under the impression of colon tumor, she was admitted for Robot Assisted Colectomy.

體檢發現

-
  - General appearance :
    - easy looking , conscious clear , without cardiopulmonary distress
    - Vital signs自動帶入date 體溫(BT) 脈搏(P) 呼吸(R) 血壓(BP) 身高(BH) 體重(BW) BMI
      20210512 35.8C 98/min 18/min 143/91mmHg 154cm 49.6kg 20.9
    - Vital signs: B.P. 136/94 mmHg , P.R. 108 /min , R.R. 18 /min , B.T. 37 ℃
  - HEENT :
    - Eyes: no anemic conjunctiva, no icteric sclera, normal EOM
    - ENT: no otorrhea, no rhinorrhea, no erythematous mucosa, no injected tonsil, no deformity
  - Neck: supple, freely movable, trachea midline,no deformity, no engorged jugular vein , no palpable thyroid or lymph node enlargement
  - Chest:
    - Inspection: Grossly normal, symmetric and fully expansion, no accessory muscle used, no operation scar, no spider angiomata, no gynecomastia
    - Palpation: no palpable mass or nodule, no subcutaneous emphysema, no tenderness
    - Ausculation: clear breathing sound
  - Heart:

-
  - Percussion: normal heart size
  - Ausculation: regular heart beat, normal S1, S2, no gallop, no murmur
- Abdomen:
  - Inspection: in
    - flat contour, symetrically, no scar, no stria, no dilated veins, no rashes
  - Ausculation: normoactive bowel sound, no abdominal bruits
  - palpation: soft, no muscle guarding or rigidity, no local tenderness, no rebounding pain, no palpable mass, no palpable pulsation
  - Percussion: no shifting dulllness, no hepatomegaly, no splenomagaly
-

Peripheral pulsation :	CA	BA	RA	FA	PA	PTA	DPA
Right side :	++	++	++	++	++	++	++/+++
Left side :	++	++	++	++	++	++	++/+++

- Genito-anus: no tenderness or palpable mass lesion or rectal shelf, normal external genitalia, normal anal tone, brown stool

CRS CONDITION

-
  - Lung

手術日期及方法

- 手術日期及方法20210513 Robot Assisted Colectomy(R)

住院治療經過

- After admission, Robot Assisted right hemicolectomy was done smoothly on 2021/05/13 under no contraindication. Under stable condition, she was discharge and arranged OPD followed up.

合併症

一般檢查

- 累積報告 :

DATE	TIME	ALB	TP	BIL.T	BIL.D	ALKP	AST	ALT	LDH	CHOL	TG	HDL-C	LDL	UA	NH3	AMY
1100513	1950	3.0		0.49	0.12	49	18	24								

DATE	TIME	WBC	RBC	HGB	HCT	MCV	PLT	BLST	NEUT	BAND	SEG	LYM	MON	EOS	BAS
1100512	2140	4520	4.37	13.0			221		54.4			35.4	5.8	4.0	0.4
1100513	1949	9920	3.32	10.0	30.4	91.6	150		90.4			6.8	2.4	0.2	0.2

DATE	TIME	NA	K	CL	CA	BUN	CREAT	Mg	P	CRP	ESR	PCT	LIPASE	LACTATE
1100512	2142	145	3.2	107	8.9	13	0.72							
1100513	1950	144	3.4	112	6.8	10	0.65							

DATE	TIME	GLUCOSE	PAB	TRF	ZN	CU	KETONE	CK	CKMB	PT-P	PT-C	APTT-P	APTT-C	TRO-T
1100513	1950	138												

1.

特殊檢查

病理報告

其它

出院時情況

- 3.改門診治療

出院指示

-

藥名	劑量	劑量單位	用法	頻次	數量	數量單位	現況	天數	單位
Mag. Oxide tab	2	TAB	PO	TID	84	TAB	TH	14	天
Celebrex cap 200mg	1	CAP	PO	QD	14	CAP	TH	14	天
BethanechoL tab 25mg-JS	1	TAB	PO	TIDAC	21	TAB	TH	7	天

(□)Takepron OD tab 30mg	1	TAB	PO	QDAC	14	TAB	TH	14	天
$Biofermin tab	2	TAB	PO	TID	96	TAB	TH	16	天

出院計劃

主治醫師 2062F 蔣鋒帆 / 住院醫師 9760F 趙英如

正式摘要(**電子簽章：000000217116140)**

W95 / W95

doctorRank:B
